# Implementation and impact analysis of a clinical pharmacy ambulatory: a two-year experience

**DOI:** 10.3389/fphar.2025.1706056

**Published:** 2025-10-23

**Authors:** Daniele Mengato, Laura Camuffo, Federica Todino, Luisella Cordiano, Domenica Condello, Catia Bucciol, Franca Benini, Francesco Paolo Russo, Alberto Cipriani, Stefano Sartori, Giacomo Sarzo, Michele Tessarin, Francesca Venturini

**Affiliations:** ^1^ Hospital Pharmacy Unit, Azienda Ospedale-Università Padova, Padua, Italy; ^2^ Palliative Care and Pain Service-Department of Women’s and Children’s Health, University of Padua, Padua, Italy; ^3^ Multivisceral Transplant Unit, Department of Surgery, Oncology and Gastroenterology, University of Padua, Padua, Italy; ^4^ Department of Cardio-Thoraco-Vascular Sciences and Public Health, University of Padua, Padua, Italy; ^5^ Paediatric Neurology and Neurophysiology Unit, Department of Women’s and Children’s Health, University Hospital of Padua, Padua, Italy; ^6^ Department of Surgery, Hospital Sant’ Antonio, University of Padova, Padua, Italy; ^7^ General, Administrative and Health Management Unit, Azienda Ospedale-Università Padova, Padua, Italy

**Keywords:** clinical pharmacy, clinical pharmacy ambulatory, clinical pharmacy service, best possible medication history, medication review, patient-education, pharmacist

## Abstract

**Introduction:**

Clinical pharmacy services contribute to safer and more effective medication use, yet remain underdeveloped in several countries, including Italy, due to limited recognition and funding. Local initiatives are essential to demonstrate feasibility and impact.

**Objective:**

To describe the 2-year implementation of a Clinical Pharmacy Ambulatory (CPA) in a large Italian tertiary hospital, and to evaluate its contribution to medication management, adherence, and interprofessional collaboration.

**Methods:**

The CPA was established within the Hospital Pharmacy Department of the Azienda Ospedale–Università di Padova. It provided three pharmacist-led services: best possible medication history (BPMH), medication review (MR), and patient education programs (PEPs). Services were delivered on-site and through telepharmacy, integrated into the electronic medical record. Outcomes were assessed using key performance indicators, including service volumes, fulfillment rates, acceptance of recommendations, and adherence measures. A survey of healthcare professionals assessed changes in perception of pharmacists’ role.

**Results:**

Over 2 years, the CPA served more than 2,200 patients. BPMH activity increased by 12%, with fulfillment consistently above 97%. Fifty-one medication reviews generated 670 therapeutic recommendations, with high physician acceptance (over 70%). PEPs enrolled 151 patients, achieving excellent adherence outcomes. Surveys showed improved awareness of the pharmacist’s role and stronger support for their integration into multidisciplinary care.

**Conclusion:**

The CPA represents the first structured pharmacist-led ambulatory formally described in Italy. It demonstrates feasibility, clinical relevance, and organizational value in a healthcare system where clinical pharmacy is not yet institutionalized. This model may inform broader adoption of pharmacist-led services in countries where clinical pharmacy remains underdeveloped.

## Highlights

• First structured clinical pharmacy ambulatory described in Italy
• Served over 2,200 patients through reconciliation, review, and education
• Achieved high physician acceptance and excellent medication adherence
• Offers a scalable model for systems without established clinical pharmacy

## 1 Introduction

The implementation of Clinical Pharmacy Services (CPSs) remains a significant challenge in healthcare systems where clinical pharmacy is not formally recognized, as in Italy, despite being well-established in several other countries (Kilonzi et al., 2023; Elberry et al., 2022; Mekonnen et al., 2017).

Unlike countries such as the United Kingdom and the United States—where CPSs originated and clinical pharmacists are integrated members of multidisciplinary care teams—many European contexts, including Italy, are still evolving in their recognition and integration of this professional role (Carter, 2016; American College of Clinical Pharmacy, 2014).

The increasing complexity of pharmacotherapy, particularly among frail populations such as the elderly, children, and patients with chronic or rare diseases, has catalyzed local and institutional efforts to embed clinical pharmacy services into hospital practice. While some countries have advanced through national regulations others have leveraged accreditation processes or international collaborations to formalize services (Kilonzi et al., 2024; Stuhec, 2025; Guntschnig et al., 2023; Urbańczyk et al., 2023). The University Hospital of Ghent in Belgium is a notable example, where CPS implementation was strategically aligned with hospital accreditation goals (Somers et al., 2016).

Evidence from international literature consistently supports the role of CPSs in improving medication safety, optimizing pharmacotherapy, and enhancing patient outcomes, while also reducing healthcare costs by preventing medication errors and avoidable hospitalizations (Khalil et al., 2016; Morris et al., 2019; Dalton et al., 2017). Despite these demonstrated benefits, the rollout of CPSs in Italian hospitals remains fragmented, hindered by the lack of structural funding and formal recognition. However, the benefits of CPSs demonstrated in the international context in terms of improving clinical outcomes, such as medication safety, adherence, and appropriateness of pharmacotherapy, have driven the development of such initiatives (Somers et al., 2016; Khalil et al., 2016; Morris et al., 2019; Dalton et al., 2017; Dooley et al., 2004).

Nevertheless, local initiatives are emerging to demonstrate the clinical and organizational value of CPSs. One such initiative is the Clinical Pharmacy Ambulatory (CPA) at Padova University Hospital (Azienda Ospedale–Università di Padova, AOUP), developed to address key pharmacotherapeutic challenges through a structured outpatient and inpatient ambulatory model. The CPA was implemented using existing institutional resources from the Hospital Pharmacy Department, without dedicated external funding. Pharmacists involved were part of the department’s permanent staff and performed CPA activities as part of their assigned duties.

This manuscript describes the development, implementation, and evaluation of the CPA at AOUP, with a focus on its three core services: Best Possible Medication History (BPMH) collection, medication review (MR), and patient education programs (PEPs). It also presents outcome data from the clinic’s first 2 years of operation and discusses implications for the future development of CPSs in hospital settings.

We followed the Standards for Quality Improvement Reporting Excellence (SQUIRE) Reporting Guideline in order to present our initiative in a standardized format (Ogrinc et al., 2016).

## 2 Aim

This project aimed to describe the 2-year implementation of a pioneering CPA service at a tertiary-care academic hospital in Italy. Specifically, the objectives were to:

• Describe the development and integration of the CPA model within the hospital pharmacy department of the AOUP, including its structure, staffing, operating procedures, and service portfolio.
• Evaluate the impact of the CPA services - including BPMH collection, comprehensive MR, and development of PEPs - on patient care processes, medication optimization, and interprofessional collaboration.
• Identify key enablers and barriers to the integration and scalability of CPS within existing clinical pathways in a setting and in a national outlook where clinical pharmacy is not yet fully institutionalized.

The analysis provides insight into practical implementation strategies and outcomes, offering a potential model for replication in similar healthcare contexts.

## 3 Methods

### 3.1 Setting

The CPA was developed and implemented in January 2023 at the AOUP, one of the largest academic hospitals in Italy, with over 1,700 beds and 101 specialized units, including the Hospital Pharmacy (HP) Department. The hospital integrates advanced clinical care, education, and research, serving as a national and regional referral center.

The CPA is embedded within the HP, which includes 19 pharmacists and six specialized areas of interest, including one dedicated to clinical pharmacy (CP). The CP area was established in 2023 to meet growing institutional needs related to medication safety, pharmacotherapy optimization, and patient engagement in therapeutic management, especially for frail or complex patient populations. The department leader identified these areas where pharmaceutical expertise could significantly impact patient care, focusing on medication reconciliation, review, and patient education.

The CPA represents an innovative model of structured, proactive pharmaceutical care delivery in an Italian hospital setting, where clinical pharmacy is not yet fully recognized within the national reimbursement framework.

### 3.2 Intervention: clinical pharmacy ambulatory model implementation

The CPA delivers three pharmacist-led services:

• BPMH collection–designed to reduce medication discrepancies during care transitions, particularly in patients with scheduled admissions. Using telepharmacy, pharmacists conduct structured interviews with patients or caregivers before admission. Medication histories are reconciled with existing electronic records and uploaded into the hospital’s electronic medical record (EMR) system, enabling informed prescribing decisions by the care team.
• MR–performed mainly at physician request for patients with polypharmacy or complex pharmacotherapy. Following BPMH collection, pharmacists evaluate indications, dosing, interactions, adverse effects, deprescribing opportunities, and adherence barriers. Recommendations are documented in standardized forms and communicated directly to prescribers. Validated tools are applied, adapted when necessary to pediatric or geriatric populations.
• Patient education programs (PEPs) – pharmacist-initiated, targeted interventions for therapies associated with high complexity, new administration techniques, or documented adherence challenges. Educational sessions include disease- or drug-specific content delivered through brochures, demonstration kits, or digital material. Current PEPs include support programs for patients treated for hepatitis D (HDV) and transthyretin cardiac amyloidosis (ATTR-CM) while a third PEP for patients with hematological malignancies has started in the early 2025 (Mengato et al., 2024) Education is delivered in-person or via telepharmacy and addresses administration technique, adherence strategies, safety monitoring, and lifestyle considerations.

The CPA is staffed by 2.0 full-time equivalent (FTE) senior clinical pharmacists, 0.5 FTE pharmacy residents, and 1.0 FTE pharmacy students. Activities are integrated into the AOUP digital ecosystem through the EMR, ensuring traceability and visibility of all interventions. Standard operating procedures regulate eligibility, scheduling, documentation, and communication.

### 3.3 Procedures and tools

Pharmacists are trained in medication reconciliation, pharmacotherapy review, and counseling, with continuous professional development in key therapeutic areas. Clinical activities are supported by multiple tools:

• EMR and hospital scheduling systems;
• validated interview templates and documentation formats;
• national drug databases and local formularies (with pediatric appropriateness checks);
• clinical decision support systems (CDSS) for drug–drug interaction detection and therapeutic monitoring.

### 3.4 Evaluation framework

The implementation of the CPA followed the quality-improvement principles promoted by the Hospital’s Internal Quality Office, which coordinates initiatives based on the Plan–Do–Study–Act (PDSA) model. Accordingly, the Quality Office recommended defining measurable Key Performance Indicators (KPIs) to guide continuous monitoring and improvement. The KPIs developed in this context were chosen for their measurability, feasibility, and managerial relevance, and are consistent with the hospital’s Quality Management System (Table 1). Data were collected via automated dashboards (Qlikview^®^, King of Prussia, PA, US) manual audits, and the EMR. Pharmacists were responsible for data entry and accuracy. Year-to-year variation was assessed using percentage change (Δ%) between 2023 and 2024.

• For BPMH: total number of services performed and requested (with relative percentages), patients served, fulfillment rate (defined as the proportion of service requests successfully delivered out of total requests received).
• For MR: number of reviews, pharmacist recommendations, and acceptance rates.
• For PEPs: service requests, number of sessions, patient enrollment, and adherence outcomes. Adherence was assessed using medication possession ratio (MPR) and proportion of days covered (PDC) (Canfield et al., 2019)
• Professional perception: a structured survey adapted from Omar et al. (Omar et al., 2020) was administered to physicians and nurses from the pediatric department, where both BPMH collection and the MR service were implemented, to capture perceptions of pharmacist integration and interprofessional collaboration within this initial implementation setting. The questionnaire used a 5-point Likert scale (from “strongly disagree” to “strongly agree”) and was administered before and 6 months after CPA implementation. For clarity of presentation, only the proportion of respondents selecting “I agree” or “strongly agree” is reported in the results.

**TABLE 1:** Overview of Key Performance Indicators (KPIs) applied to the three main activities of the Clinical Pharmacy Clinic (Best Possible Medication History [BPMH] collection, Medication Review, and Patient Education Programs [PEPs]). Each KPI is classified according to its applicability to the specific service area.

KPI number	KPI description	BPMH collection	Medication review	PEPs development
1	Number of services provided	Yes	Yes	Yes
2	Number of requesting clinical units/department	Yes	Yes	Yes
3	Number of patients served	Yes	Yes	Yes
4	% of services provided out of total requests (target ≥90%)	Yes	Yes	Yes
5	% of pharmacist recommendations accepted (target ≥40%)	Not applicable	Yes	Not applicable
6	% of patients adherent to therapy following PEP (target ≥90%, measured by standardized methods - Proportion of Days Covered [PDC] and/or Medication Possession Ratio [MPR])	Not applicable	Not applicable	Yes

The evaluation framework was designed both to guide continuous internal quality improvement and to provide external evidence supporting scalability of the CPA model.

### 3.5 Data analysis

Data from EMR, automated dashboards, and manual audits were compiled to calculate KPIs. Service volumes, fulfillment rates, and recommendation acceptance were summarized using descriptive statistics. Adherence was assessed through MPR and PDC. Survey responses from healthcare professionals were analyzed descriptively, and changes between baseline and follow-up were examined using chi-square or Fisher’s exact tests, when appropriate. Statistical analyses were performed using standard software packages, with significance set at p < 0.05.

## 4 Results 2 years after ambulatory opening

After 2 years of operation, the CPA at AOUP has demonstrated significant achievements across its three main service areas. The results reflect both the volume of services provided and their impact on patient care and medication safety. Notably, requests for BPMH and MR services were typically submitted by physicians or clinical units through the hospital’s electronic system, whereas PEPs were proactively initiated by pharmacists in agreement with the prescribers, based on therapy complexity and patient needs.

Subsequent subchapters of this section will present the results, split by the three activity areas, achieved in the years 2023 and 2024, with related trends. For each area, applicable KPIs will be used as indicators.

### 4.1 Best possible medication history collection results

Since its launch in the early 2023, the CPA performed a total of 2,050 BPMHs across seven clinical units, in surgery and pediatrics area. Activity increased by 12% between 2023 and 2024, reflecting progressive integration into preadmission workflows. Fulfillment rates remained consistently high, above 97% overall. Surgical units achieved near-complete fulfillment (100%), while pediatric services showed slightly lower values (from 92% to 85%) due to urgent or short-notice admissions. These findings demonstrate strong feasibility of the telepharmacy-supported BPMH model. For further information, detailed by the two areas, see Table 2.

**TABLE 2:** Comparative analysis of BPMH service performance across surgical and pediatric clinical areas in 2023 and 2024, showing total BPMHs provided and requested, patients served, and units involved, with percentage change (Δ%) between the 2 years.

Clinical areas	KPIs	2023	2024	Δ% 2024 vs. 2023
Surgical Units	Total BPMH provided	791	895	13%
	Total BPMH requested	791	895	13%
	Total patients served	772	881	14%
	Total units involved	3	3	0%
Pediatric Units	Total BPMH provided	173	191	10%
	Total BPMH requested	189	226	20%
	Total patients served	144	152	6%
	Total units involved	3	4	33%

### 4.2 Medication review service results

The MR service was initiated in late 2023 and piloted in three pediatric units. Over the evaluation period, 51 reviews were conducted for 50 patients, generating 670 pharmacist recommendations. The overall fulfillment rate was 98.1%. Recommendation acceptance varied by setting: outpatient reviews reached 97.5% acceptance, while inpatient reviews reached 71.6%. Most accepted interventions related to prevention of potential medication errors (84%) and optimization of dosage or formulation (80%). Lower acceptance was observed for logistical recommendations such as drug handling or prescribing processes. These data indicate strong clinical relevance of pharmacist interventions, particularly in outpatient care (Table 3).

**TABLE 3:** Summary of Medication Review activity by care setting (inpatients vs. outpatients), including the number of reviews performed, pharmacist suggestions provided, suggestions accepted by physicians, and acceptance rate (%).

Care setting	Medication reviews performed	Suggestions given by pharmacists	Suggestions accepted by physicians	% Of accepted suggestions
Inpatients	9	194	139	71.6%
Outpatients	42	476	464	97.5%

### 4.3 Patient education programs (PEPs) development results

Two pharmacist-led PEPs were launched during the study period, targeting patients treated with tafamidis for transthyretin cardiac amyloidosis and bulevirtide for chronic hepatitis D virus infection. In total, 151 patients were enrolled, accounting for 339 education encounters. Each dispensing event served as an opportunity for structured education, with initial visits averaging 20 min and follow-up visits 10 min.

Both programs expanded rapidly: the cardiology PEP grew from 31 sessions in 2023 to 68 in 2024 (+113%), while the hepatology PEP increased from 67 to 173 sessions (+258%). Importantly, 100% of service requests were fulfilled. Adherence outcomes exceeded predefined targets: all patients with hepatitis D achieved PDC ≥80%, and 98.5% of patients treated with tafamidis achieved MPR ≥80%. A third PEP, focusing on hematological malignancies and oral targeted therapies, was introduced in early 2025, with results forthcoming. Table 4 outlines the clinical and logistical drivers that inspired the development of these programs in their respective therapeutic areas.

**TABLE 4:** Drivers for the Implementation of Patient Education Programs (PEPs), clusterized by program.

Justification/Driver	HDV - bulevirtide	Amyloidosis – Tafamidis
High complexity in drug storage, preparation, and administration and need for training on self-administration or handling procedures	Yes	Not-applicable
Therapeutic adherence crucial to drug effectiveness and safety	Yes	Yes
Novel drug on market: need for real-world data on effectiveness and safety	Yes	Yes
High cost of the drug	Yes	Yes
Patient population with complex comorbidities or special support needs	Not-applicable	Yes
Risk of poor adherence due to asymptomatic or insidious nature of the disease	Yes	Not-applicable
Only available therapeutic option in national formulary	Yes	Yes
Multiple prescribers in AOUP: need for a harmonized approach	Yes	Not-applicable
Need to collect Patient Reported Outcomes (PROs)	Yes	Yes
Drug under special monitoring for safety: proactive pharmacovigilance project	Yes	Yes

### 4.4 Feedbacks on the value of the CPA from other health professionals

A total of 60 pediatric healthcare professionals (physicians and nurses) completed the perception survey at baseline (T0) and 6 months after CPA implementation (T1). Positive responses increased across all 12 items, with significant improvements for three statements: awareness of the clinical pharmacist’s presence (p < 0.05), belief that pharmacists improve care quality (p < 0.05), and recognition of pharmacists in the workplace (p < 0.05). Table 5 highlights that although baseline familiarity with clinical pharmacists was limited, perceptions of their impact were highly positive, indicating that exposure to CPA services rapidly improved recognition and collaboration. Subgroup analysis by profession was not feasible due to sample size but will be considered in future surveys. These findings confirm that the CPA not only impacted patient-level outcomes but also enhanced the visibility and integration of pharmacists within multidisciplinary teams.

**TABLE 5:** Results of the survey evaluating healthcare staff perceptions regarding the role of the clinical pharmacist, comparing responses at baseline (T0) and after 2 years of Clinical Pharmacy Clinic activity (T1). Data are expressed as the percentage of “I agree” responses; p-values assess the statistical significance of observed changes.

Question number	Question	“I agree” or “strongly agree” answer at t0 N = 67 (%)	“I agree” or “strongly agree” answer at t1 N = 60 (%)	p value
1	Healthcare staff are used to collaborate with the clinical pharmacist	18 (26.9)	27 (45.0)	0.08
2	The clinical pharmacist can improve patient care	62 (92.5)	60 (100)	0.12
3	The clinical pharmacist is an important figure in the ward team	51 (76.1)	49 (81.7)	0.50
4	Clinical pharmacist services have or will have a positive impact on pediatric patient management	62 (92.5)	55 (91.7)	>0.09
5	The clinical pharmacist in the ward team improves care activities	57 (85.1)	54 (90.0)	0.4
6	The clinical pharmacist is able to reduce therapeutic errors and improve patient therapy outcomes	64 (95.5)	58 (96.7)	0.9
7	I have already heard of the clinical pharmacist at my workplace	44 (65.7)	51 (85.0)	<0.05
8	The presence of the clinical pharmacist in a ward team would improve the quality of patient care in the hospital setting	45 (67.2)	52 (86.7)	<0.05
9	The clinical pharmacist has a role in the pharmacological education of the patient	59 (88.1)	57 (95.0)	0.4
10	The clinical pharmacist is present at my workplace	35 (52.2)	53 (88.3)	<0.05
11	It would be useful for the clinical pharmacist to acquire specific training for the clinical field in which they operate	62 (92.5)	58 (96.7)	0.7
12	The presence of the clinical pharmacist during ward rounds and in collegial discussions is desirable	62 (92.5)	59 (98.3)	0.4

## 5 Discussion

This study described the implementation and 2-year evaluation of a CPA in a large Italian tertiary hospital. The findings demonstrate that pharmacist-led services can be successfully integrated into routine hospital practice even in settings where clinical pharmacy is not formally recognized at the national level (Wang et al., 2025; Abdulghani et al., 2018). The CPA achieved consistent growth, high acceptance of pharmacist recommendations, excellent adherence outcomes, and improved professional recognition, serving more than 2,200 patients through medication reconciliation, medication review, and patient education.

The results are consistent with international evidence highlighting the positive impact of clinical pharmacy services on medication safety, therapy optimization, and patient outcomes. (Stuhec, 2025; Guntschnig et al., 2023; Somers et al., 2016; Khalil et al., 2016; Morris et al., 2019). In particular, the high acceptance of pharmacist recommendations mirrors findings from other European and North American experiences, where integration of pharmacists into clinical teams has been associated with improvements in prescribing quality and patient safety (Carter, 2016; American College of Clinical Pharmacy, 2014; Somers et al., 2016). The strong adherence outcomes observed in our patient education programs also align with previous reports emphasizing the role of pharmacist-led counseling in supporting the use of complex or high-cost therapies (Mengato et al., 2024).

From a policy perspective, this experience is particularly relevant for Italy, which currently lacks a formal framework for clinical pharmacy services, leaving their adoption fragmented and dependent on local initiatives (Lombardi et al., 2018; Mengato et al., 2023; Fornasier et al., 2018). The CPA provides the first structured and documented example of a clinical pharmacy ambulatory in the country, demonstrating feasibility and measurable impact. Its success supports calls for broader institutional recognition of clinical pharmacists and for systematic inclusion of CPSs in multidisciplinary care pathways.

At the European level, this case contributes to the evidence base for countries where clinical pharmacy remains underdeveloped or insufficiently institutionalized (Stuhec, 2025; Guntschnig et al., 2023). The CPA model, characterized by standardized procedures, digital integration, and outcome monitoring through key performance indicators, offers a transferable framework for scaling similar services in healthcare systems with limited or emerging CPS structures. Demonstrating both clinical and organizational value is essential to secure sustainable funding and to embed pharmacists within care teams, thereby aligning national policies with international best practice.

Moreover, these results reflect interprofessional perceptions generated by exposure to CPA activities (BPMH and MR) in the pediatric teams, and suggest that early, visible pharmacist involvement can improve recognition of pharmacists’ contributions and facilitate collaborative care.

This study also has strengths and limitations. A major strength lies in the comprehensive scope of the CPA, integrating medication reconciliation, review, and patient education within a single service, and in the use of standardized indicators for monitoring. Integration into the electronic medical record ensured visibility and traceability of all interventions. However, being a single-center experience limits generalizability, and the short observation period restricts evaluation of long-term outcomes. In addition, while clinical and process outcomes were captured, the economic impact of the model has not yet been assessed.

Looking ahead, the CPA will consolidate existing programs and expand into new therapeutic areas, such as hematology. A key future priority will be the evaluation of the economic impact of the model, including potential cost savings from error prevention, improved adherence, and more efficient use of high-cost therapies (Baudo et al., 2021). Demonstrating financial sustainability is expected to strengthen the case for long-term institutional support and board-level adoption.

Implementation outcomes were also evaluated following Proctor’s taxonomy, focusing on acceptability, feasibility, fidelity, and sustainability (Proctor et al., 2011). Acceptability was reflected by high physician acceptance of pharmacist recommendations; feasibility was demonstrated by consistent service growth and high fulfillment rates; fidelity was supported by standardized procedures and integration into the electronic medical record; and sustainability is being pursued through institutional recognition and alignment with hospital quality improvement goals. Future work will explore additional implementation outcomes such as penetration and cost-effectiveness.

Our evaluation approach, developed with the Hospital’s Internal Quality Office, was inherently aligned with the PDSA model that governs institutional quality-improvement activities. Using KPIs consistent with this framework allowed us to capture key process and outcome dimensions (acceptability, feasibility, fidelity, and sustainability). In future phases, we plan to integrate formal implementation frameworks such as CFIR, RE-AIM, and EPIS to systematically assess contextual determinants and long-term adoption (Damschroder et al., 2022; Holtrop et al., 2021; Moullin et al., 2019).

It is also important to give adequate attention to facilitators and barriers. Key drivers of successful implementation included institutional endorsement from the Internal Quality Office, alignment with the PDSA cycle, and seamless integration of activities within the EMR. The main barriers were limited personnel resources and the absence of protected time for clinical pharmacy activities in some departments.

To strengthen implementation and scalability, future strategies should focus on formalizing interprofessional communication processes, expanding CPA coverage to additional wards, embedding digital referral and follow-up tools within the EMR, and incorporating CPA activities into staff training and performance evaluation systems. These measures would consolidate the long-term sustainability of the model and facilitate broader institutional adoption.

In summary, the CPA represents a pioneering and scalable model of pharmacist-led care that enhances medication management, supports adherence, and fosters interprofessional collaboration. Its early success highlights the importance of local innovation in advancing clinical pharmacy in contexts lacking national recognition. This Italian experience may serve as a reference for other European healthcare systems seeking to institutionalize clinical pharmacy services as a core component of patient-centered care.

## 6 Conclusion

The CPA established at a large Italian tertiary hospital represents the first structured model of its kind formally described in Italy, demonstrating that pharmacist-led services can be integrated successfully into hospital workflows, achieving measurable benefits in medication safety, adherence, and interprofessional collaboration.

This experience shows that clinical pharmacy can generate value even in healthcare systems where it is not formally recognized, providing a transferable framework for other countries in which clinical pharmacy services remain underdeveloped. By standardizing processes, embedding activities in digital platforms, and monitoring outcomes through predefined indicators, the CPA has proven both feasible and impactful.

Future priorities include evaluating the economic implications of the model to demonstrate cost savings from improved adherence and safer use of high-cost therapies. Such evidence will be crucial to support institutionalization, secure sustainable funding, and inform national policy. The CPA thus offers a pioneering example of how local innovation can catalyze broader adoption of clinical pharmacy services, contributing to more patient-centered and efficient healthcare delivery.

## Data Availability

The raw data supporting the conclusions of this article will be made available by the authors, without undue reservation.
